# Decompression with fusion is not in superiority to decompression alone in lumbar stenosis based on randomized controlled trials: A PRISMA-compliant meta-analysis

**DOI:** 10.1097/MD.0000000000017849

**Published:** 2019-11-15

**Authors:** Shuai Xu, Jinyu Wang, Yan Liang, Zhenqi Zhu, Kaifeng Wang, Yalong Qian, Haiying Liu

**Affiliations:** a Department of Spinal Surgery, Peking University People's Hospital, Peking University, Beijing; b Department of Spinal and Joint Surgery, Taishan Medical University Affiliated Qingdao Hospital, Taishan Medical University, Qingdao, Shandong, P.R. China.

**Keywords:** decompression alone, decompression with fusion, degenerative spondylolisthesis, lumbar spinal steonsis, meta-analysis

## Abstract

**Background:**

Although some studies had been published, it was more controversial on the superiority of decompression alone (D) and decompression with fusion (F) for the treatment of lumbar spinal stenosis (LSS) recently, especially newest articles with different opinions. A meta-analysis was performed to compare efficacy on D and F for LSS regardless of degenerative spondylolisthesis (DS) with randomized controlled trials (RCTs).

**Methods:**

The databases include PUBMED/MEDLINE, EMBASE, Cochrane Library, and Web of Science from January 1970 to December 2018. The information of screened studies included demographics, clinical outcomes, and secondary measures, then data synthesis and meta-analysis were progressed. Subgroup analysis was stratified by DS and follow-up time (36 months). Continuous variables and dichotomous variables were respectively reported as weighted mean difference and odds ratios (ORs). The strength of evidence was evaluated by the grades of recommendation, assessment, development, and evaluation (GRADE) system.

**Results:**

Nine RCTs met inclusion criteria with a total of 857 patients (367 were in D group and 490 were in F group). There were no statistical difference in visual analog scale changes on back and leg pain between D and F group (mean difference [MD] = −0.03, 95% confidence interval [CI] [–0.38, 0.76], *z* = 0.08, *P* = .94; MD = 0.11, 95% CI [–1.08, 1.30], *z* = 0.18, *P* = .86, respectively); patients’ satisfaction was of no difference between the 2 groups, together with the change of the Oswestry disability index and European quality of life-5 dimensions (*P *>* *.05). There were no difference in the rate of complication (*P* = .50) and reoperation (*P* = .11) while a statistical significance of longer operation duration (*P* < .0001), more blood loss (*P* = .004) but amazing lower rate of adjacent segment degenerative/disease (ASD) (OR = 2.35, *P* = .02) in F group. The subgroup analysis on DS showed that basically all measures were in consistency with meta-analysis. There was a higher reoperation rate in middle-to-long term (>36 months) in D group and ASD was the most seasons of reoperation no matter the follow-up time. According to the GRADE system, the grade of this meta-analysis was of “High” quality.

**Conclusion:**

F group has no better clinical results than D alone in LSS, regardless of DS and follow-up. The conclusion is of “High” quality and the grade strength of recommendation was “Strong.”

## Abstract 2

Supplemental Digital Content is available in the text

## 1 Introduction

Lumbar spinal stenosis (LSS) is the most common disease in vertebrae or disc lumbar spondylosis, which is characterized by narrowing of the central vertebral canal, lateral recesses.^[1–2]^ Degenerative spondylolisthesis (DS), approximately 4.1% in general population^[3]^ and usually accompanied with LSS, due to degenerative changes resulting in slip of 1 vertebral body over another, causing a series of symptoms of intermittent neurogenic claudication, radicular back, and leg pain. The therapy strategy has been identified that surgical intervention was superior to conservative care for symptomatic lumbar spondylosis by the spine patient outcomes research trial.^[4]^

Decompression (D) is a recommended surgical approach of LSS and D with fusion (F) is even regarded as the gold standard surgery on DS for the stability support.^[5]^ However, the issue on whether fusion is absolute need remains still controversial.^[6–8]^ Over the last 2 decades, several reviews on comparison of surgical outcomes between D alone and D plus F for LSS have been published and some of them are in favor that F had better clinical outcomes.^[3,9–10]^ However, with the publish of qualified randomized controlled trials (RCTs) about D and F of LSS drawing somewhat different conclusion, the opinion on this focus progressed more controversial.

Therefore, a meta-analysis is still of vital importance to be performed since the lack of qualified study consist of non-randomized controlled trials (nRCTs), the neglect of data published by Forsth et al in 2017,^[11]^ the paucity of evidence on all outcomes but the primary ones, the lack of grades of recommendation on the whole meta-analysis. Therefore, we conducted a meta-analysis and systematic reviews to compare the entire efficacy on D with F for patients with 1- to 2- level LSS (with or without DS) based on published RCTs.

## 2 Methods

### 2.1 Search strategies

An ethics committee of the Peking University People's Hospital approved the study. The databases used to search include PUBMED/MEDLINE, EMBASE, Cochrane Library, and Web of Science for English-language articles, from January 1970 to December 2018. The following search strategies were used: (laminotomy OR laminectomy OR fenestration OR hemilaminectomy OR decompression) AND (lumbar spondylolisthesis OR lumbar spinal stenosis OR lumbar canal stenosis OR degenerative lumbar spondylolisthesis OR slipped disk OR protrusion OR herniated disc) AND (fusion OR arthrodesis). Two reviewers independently screened all studies for eligibility.

### 2.2 Inclusion and exclusion criteria

Included studies fulfilled the following criteria:

(1) they were RCTs written in English;
(2) the studies focused on the comparison between D versus F for LSS and (LSS combined with hernia disc syndrome) HD, the LSS was with or without DS;
(3) the comparative data of clinical outcomes, major complications, reoperations, and other perioperative desirable outcomes could be acquired, and
(4) the sample size was bigger than 5 per group and a minimum follow up time of 1 year.

Exclusion criteria were:

(1) non-English-language articles;
(2) nRCTs, case reports, duplicate papers, or review reports;
(3) without a controlled group or with a small sample size (<5 patients per group);
(4) participants mixed tumors, fractures, osteoporosis, or other irrelevant diseases;
(5) studies mainly concerning a surgical approach, or surgical techniques or instruments;
(6) studies with incomplete or undesirable outcome.

### 2.3 Data extraction

Both reviewers assessed potentially eligible trials and extracted information independently from each potential study. Any discrepancies were resolved through a third reviewer to reach consensus. The following data were extracted: basic characteristics of demographic information, primary and secondary measures. Primary measures included the change of visual analog scales (VASs, ranging from 0 to 10, with higher scores indicating more severe pain) on back and leg pain, the Oswestry disability index (ODI, ranging from 0 to 100, with higher scores indicating more disability related to pain), European quality of life-5 dimensions (EQ-5D, range ranging from 0 to 1, with higher score indicating better quality of life), medical outcomes study 36-item short-form health survey (SF-36), patients’ satisfaction, walking ability. Secondary measures included that included incidence of complications and reoperations, operation time, blood loss, length of hospitalization and adjacent segment degenerative/disease (ASD).

### 2.4 Risk of bias and quality assessment

Two investigators independently graded each eligible study. We used the Cochrane Handbook for Systematic Reviews of Interventions, version 5.0^[12]^ for RCTs. The following domains were assessed: randomization, blinding (of patients, surgeons, and assessors), allocation concealment, adequacy of outcome data, selective reporting, and other biases. Each domain of quality assessment was classified as adequate (A), unclear (B) or inadequate (C). If all domains were A, the study was A-level; if at least 1 domain was B, the study was B-level; if at least 1 domain was C, the study was C-level.

### 2.5 Data synthesis and analysis

Review Manager Software (RevMan Version 5.3 [The Cochrane Collaboration, Oxford, United Kingdom]) was used to conduct the statistical analysis.

Continuous variables were reported as weighted mean difference and 95% confidence interval (95% CI), and dichotomous variables were reported as odds ratios (ORs) and 95% CI. Results were regarded as statistically significant if 2-sided *P* < .05. *I*^2^ was used to estimate the size of the heterogeneity. *I*^2^ < 50% indicated low heterogeneity and the results of comparable groups could be pooled using a fixed-effects model. Subgroup analysis that could reduce statistical heterogeneity to facilitate factor definition was worthwhile. If the overall heterogeneity was *I*^2^ < 50%, we could still divide studies into subgroups depending on professional principles and clinical meaning.

### 2.6 GRADE approach

The grades of recommendation, assessment, development, and evaluation (GRADE) approach was used to evaluate the strength of evidence.^[13]^ Based on parameters, the quality assessment was classified as very low, low, moderate, or high according to the GRADE handbook (version 3.2), with the GRADE profiler software (version 3.6). A summary of findings table (SoF Table) was used to explain the final results.

## 3 Results

### 3.1 Search result

**Figure 1:**
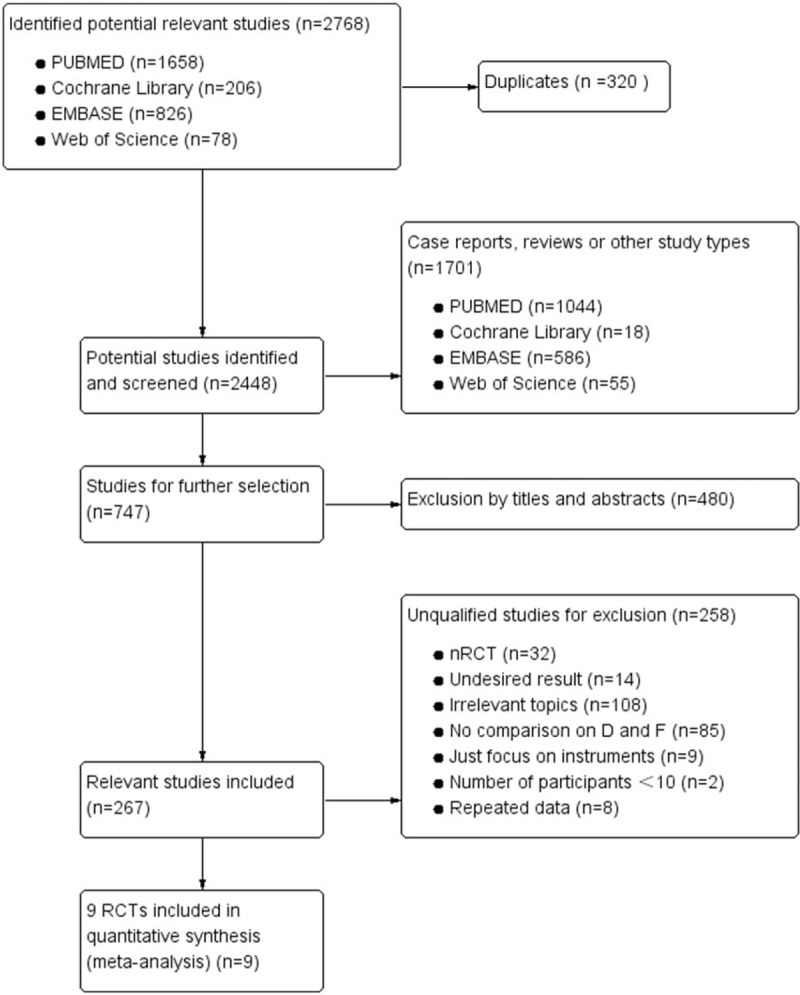
Flow diagram on selection for included RCTs. RCTs = randomized controlled trials.

The process of identifying relevant studies is summarized in Figure 1. Two thousand seven hundred sixty-eight references were obtained from the databases mentioned and a total of 9 RCTs^[14–22]^ eventually met inclusion criteria with a total of 857 patients: 367 were in D group and 490 were in F group. As some studies were continuations of previous articles, we used the latest publication to avoid duplication and the 9 included studies were published between 1987 and 2016. Two RCTs published in 2017 completed by Försth et al and Karlsson et al^[11,23]^ contained the same data as the study published in 2016, we finally could not regard the 2 RCTs as included studies but only adopt partial refreshed information as supplement for its undesirable and inadequate outcomes although published later.

### 3.2 Risk of bias and quality assessment

**Figure 2:**
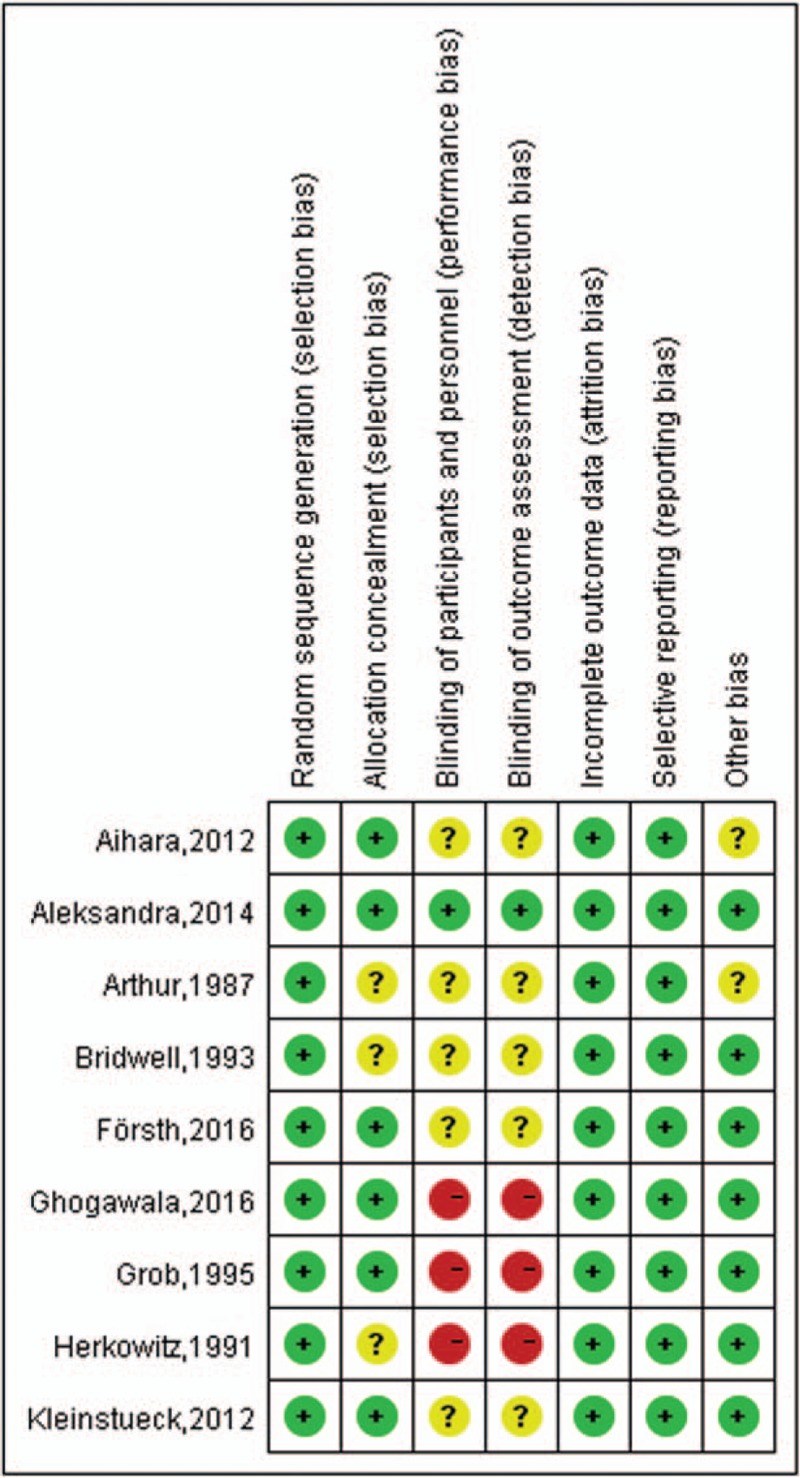
Risk of bias summary. The review authors’ judgments about each risk of bias item for each included study: + is “yes,” – is “no,” ? is “unclear.”

According to the quality assessment criteria recommended by the Cochrane Handbook for Systematic Reviews of Interventions,^[12]^ 6 out of 9 were of high quality and a low risk of bias. One study was A-level quality,^[16]^ 5 articles were B-level,^[14,17,18,20,23]^ and 3 articles were C-level with a moderate risk of bias^[15,19,21]^ (Fig. 2).

### 3.3 Results of meta-analysis

#### 3.3.1 Basic characteristics

The characteristics on basic information of the 9 included RCTs were recorded in Table 1. The participants were diagnosed with LSS combined with DS in 6 studied, LSS in 2 studies and HD in 1 study. The average age in D group and F group was of no difference (*P* = .99), so was the sex ratio (F/M) (*P* = .47). Surgery approaches in D group referred to decompression alone, laminectomy and facetectomy, while in F group contained posterior lumbar interbody fusion (PLIF), posterolateral fusion (PLF), and facet arthrodesis with or without instruments. There were of no significance on preoperative VAS on back and leg pain between the 2 groups supported by 5 articles.^[14,16–18,21]^

**Table 1:**
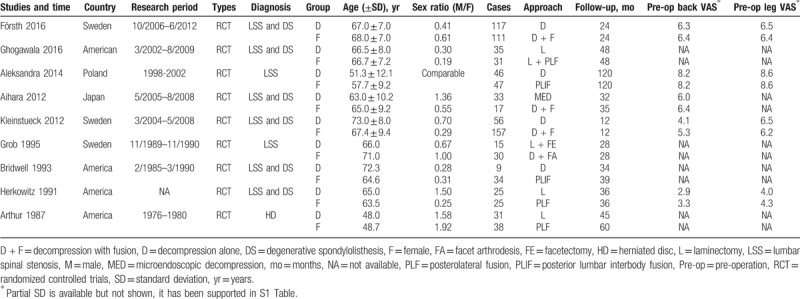
Characteristics and surgery information of the included studies.

#### 3.3.2 Primary measures

##### 3.3.2.1 VAS change on back pain

**Figure 3:**
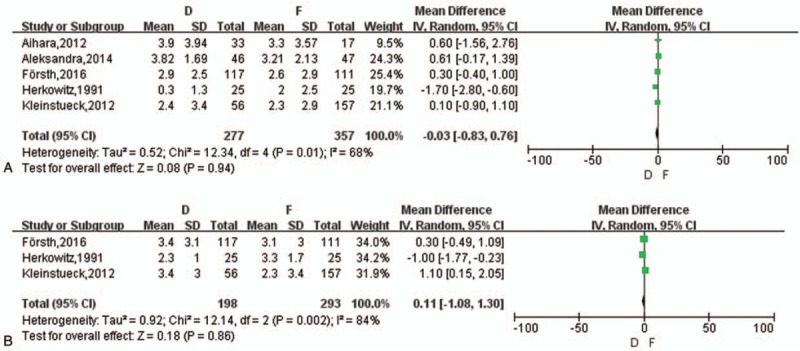
(A) The meta-analysis on the change of VAS on back pain between D and F group. (B) The meta-analysis on the change of VAS on leg pain between D and F group. VAS = visual analog scale.

Six studies^[14,16–19,21]^ reported VAS change of back pain between the 2 groups but 1 study failed to give specific value. A random-effects model was applied for meta-analysis (*I*^2^ = 68%) and there was no statistical difference in VAS changes between pre- and postoperative back pain between the 2 groups (mean difference [MD] = −0.03, 95% CI [−0.38, 0.76], *z* = 0.08, *P* = .94) (Fig. 3A). Grob et al^[19]^ reported there was of no difference between D group and F group but both amelioration contrasted with that of reoperation though a lack of precise data. The number of improvement on back pain mentioned in 3 articles^[14,18,19]^ showed no difference between the 2 groups (OR = 0.75, *z* = 1.27, *P* = .21), see Word 1, Supplemental Content, which illustrated VAS decrease on back.

##### 3.3.2.2 VAS change on leg pain

Four studies^[14,18,19,21]^ reported VAS change of leg pain and 1 study still miss specific data. A random-effects model was applied (*I*^2^ = 84%) with no difference in VAS changes between pre- and postoperative leg pain between the 2 groups (MD = 0.11, 95% CI [–1.08, 1.30], *z* = 0.18, *P* = .86) (Fig. 3B). Grob et al^[19]^ also reported there was no difference between D group and F group but both improved postoperatively. The number of improvement on leg pain mentioned in 2 articles^[14,19]^ showed no difference between the 2 groups (OR = 1.79, *z* = 0.50, *P* = .62) and 1 article^[18]^ reported no significance without specific data, see Word 2, Supplemental Content, which illustrated VAS decrease on leg.

##### 3.3.2.3 The change of ODI, EQ-5D, and SF-36

**Figure 4:**
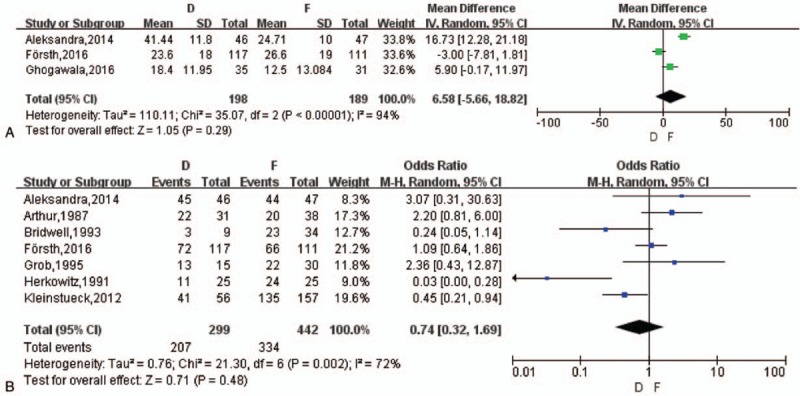
(A) The meta-analysis on the change of ODI between D and F group. (B) The meta-analysis on patients’ satisfaction between D and F group. ODI = the Oswestry disability index.

Three studies^[14–16]^ and 2 studies^[14,15]^ referred to the change of ODI and EQ-5D, respectively. There was no statistical difference in ODI change postoperatively between the 2 groups (MD = 6.58, 95% CI [–5.66, 18.82], *z* = 1.05, *P* = .29) with random-effects model (*I*^2^ = 94%) (Fig. 4A) and no difference in EQ-5D change (MD = 0.03, 95% CI [–0.04, 0.10], *z* = 0.82, *P* = .41) with fixed effects model (*I*^2^ = 0%). 1 study referred to the change of SF-36 physical-component by Ghogawala et al^[15]^ which was in favor of F group originally (*P* = .046).

##### 3.3.2.4 Patients’ satisfaction and walking ability

Seven studies^[14,16,18–22]^ reported patients’ satisfaction in contrast with that of preoperation. A random-effects model was applied (*I*^2^ = 72%) with no difference in patients’ satisfaction between the 2 groups (OR = 0.74, 95% CI [0.32, 1.69], *z* = 0.71, *P* = .48) (Fig. 4B). The increased number of in walking distance were reported of the 2 studies,^[14,19]^ a meta-analysis about it showed no statistical significance (OR = 1.07, *z* = 0.09, *P* = .93) and Aihara et al^[17]^ indicated the walking ability score (4.81 vs 4.24) of no difference between D and F group, see Word 3, Supplemental Content, which illustrated walking distance.

#### 3.3.3 Secondary measures

##### 3.3.3.1 Complications and ASD

**Figure 5:**
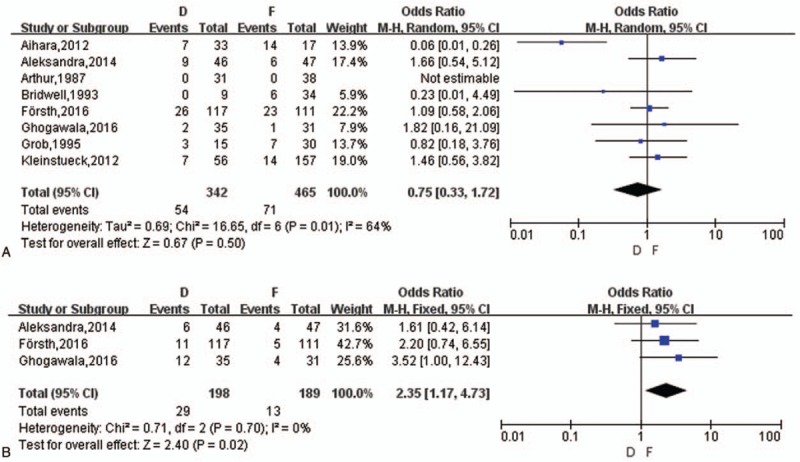
(A) The meta-analysis on complications rate between D and F group. (B) The meta-analysis on the rate of ASD between D and F group. ASD = adjacent segment degenerative/disease.

Eight studies^[14–20,22]^ reported intra- and postoperative complications (1 article reported with no complication) and 3^[14–16]^ of them mentioned ASD, which was an important outcome in follow-up postoperatively. The overall incidence of complications were 14.71% in D group and 14.49% in F group (with a range of 0% to 42%). Eventually, there was no statistical difference on complications between D group and F group (OR = 0.75, *z* = 0.67, *P* = .50) (Fig. 5A). ASD was not distinguished meticulously in this study though the different conception between adjacent segment degeneration and adjacent segment disease.^[24]^ A meta-analysis showed a difference between D group and F group (OR = 2.35, *z* = 2.40, *P* = .02) (Fig. 5B).

##### 3.3.3.2 Reoperation

**Figure 6:**
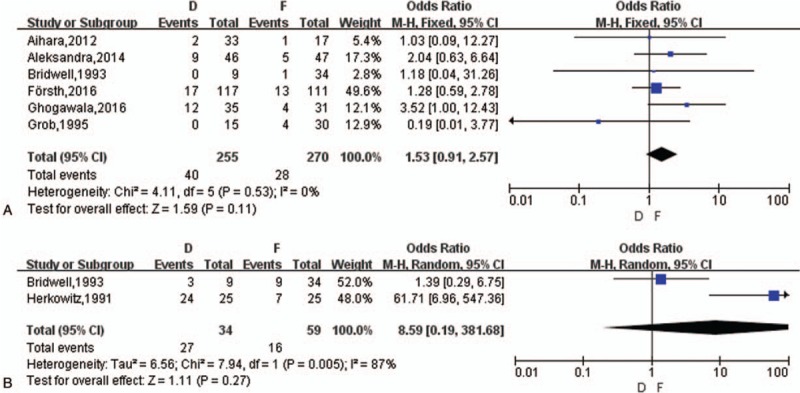
(A) The meta-analysis on reoperation rate between D and F group. (B) The meta-analysis on postoperative DS progression between D and F group. DS = degenerative spondylolisthesis.

Six studies^[14–17,19,20]^ reported reoperation and the range of reoperation rate was from 2.33% to 24.24%. ASD was the majority of reoperation in D group (72.5%), and then followed infection (15%) and recurrence of symptoms (12.5%), while the most common reason for secondary surgery in F group was also ASD (46.43%), and then were restenosis (17.86%), implantation losing or instability (17.86%), infection (10.71%) and persistent pain (7.14%). Finally, a meta-analysis showed no difference between D group and F group (OR = 1.93, *z* = 1.59, *P* = .11) (Fig. 6A).

##### 3.3.3.3 Operation duration, blood loss, and hospital stays

The duration of operation, blood loss, and length of hospital stays were simultaneously included by these 5 articles^[14–17,19]^ but 1^[19]^ out of 5 miss the standard derivation, so 4 studies could be performed meta-analysis. There was a statistical difference in operation time and blood loss between the D group and F group (MD = −80.02, *z* = 4.53, *P* < .0001; MD = −339.05, *z* = 2.86, *P* = .004, respectively) with random-effects model (*I*^2^ = 97%; *I*^2^ = 100%, respectively). Grob et al^[19]^ reported a significance on duration of operation (104 minutes vs 147 minutes) and blood loss (300 mL vs 762 mL) between the 2 groups in original article. As to the length of hospitalization, a statistical significance was also shown between the 2 groups (MD = −2.66, *z* = 4.43, *P* < .0001, *I*^2^ = 78%), see Word 4, Supplemental Content, which illustrated operation duration, blood loss and hospital stays.

##### 3.3.3.4 Postoperative DS progression

Accompanied with LSS, DS was often seen in LS and 6^[14,15,17–19,20,21]^ out of 9 included studies referred to DS with a proportion of 64.76% among selected participants. A meta-analysis on 2 studies about the number of postoperative DS progression showed no difference (OR = 8.59, *z* = 1.11, *P* = .27) (Fig. 6B). Then we performed a subgroup analysis on stratification of DS.

More details for the total meta-analysis was shown in Table, Supplemental Content, which illustrated the specific information about the overall meta-analysis.

### 3.4 Subgroup meta-analysis

#### 3.4.1 LD combined with DS

The RCT published in 2016^[14]^ reported a comparison of D group (66 patients) and F group (67 patients) with DS and other patients included 5 RCTs were all diagnosed LSS combined with DS. Bridwell et al^[20]^ showed a proportion of 26.47% occurred in L3/4 and 73.5% in L4/5 with single segment slip. Overall, the operation duration and blood loss in secondary measures were of statistical difference between the 2 groups (*P* = .004 and *P* < .0001, respectively), all of the comparisons were in consistency with the whole meta-analysis (Table 2).

**Table 2:**
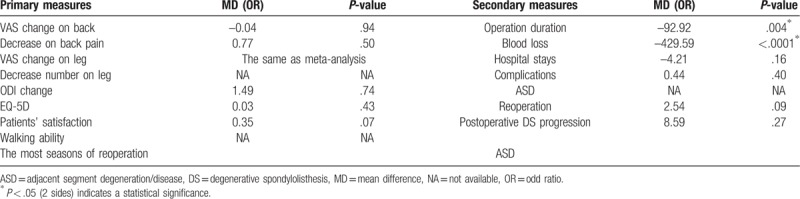
Outcomes between D and F group of subgroup analysis on DS.

#### 3.4.2 Follow up time

Long-term follow-up suggested that fusion surgery may accelerate degeneration of the adjacent segment but no influence on clinical result.^[25]^ Consequentially a subgroup analysis base on follow-up time of short term (<36 months) and middle-to-long term (>36 months) was then underwent in comparison of primary and secondary measures except operation duration, blood loss and hospital stays for their senseless. Table 3 showed that there was a statistical difference (<36 months) in VAS change on leg pain (*P* = .04) standing D group side, suggesting as least no better outcome with fusion in short term follow-up. As to the middle-to-long term follow-up, the change of ODI and reoperation rate was of significance in favor of D group and F group respectively, which indicated decompression alone may induce a higher reoperation rate with the longer follow-up. The other measures were in line with the overall meta-analysis and ASD was the most seasons of reoperation yet no matter the follow-up time.

**Table 3:**
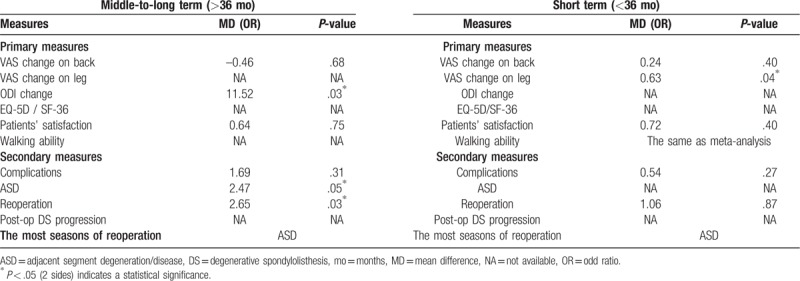
Outcomes between D and F group of subgroup analysis on follow-up time.

### 3.5 Publication bias (It can be deleted)

Publication bias was just assessed for VAS change on back pain, reoperation rate, and complications as at least 5 studies are required to detect asymmetry. Funnel plot showed no apparent asymmetry (not shown) and Egger test showed *P* > ltl = .160, suggesting that publication bias may not be a limitation.

### 3.6 GRADE approach

**Figure 7:**
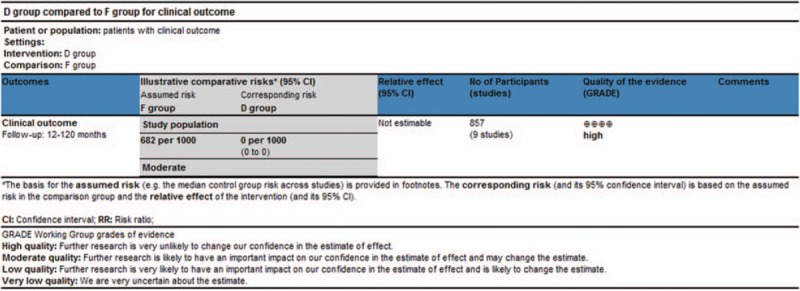
Preview SoF table of the GRADE for this meta-analysis. GRADE = the grades of recommendation, assessment, development, and evaluation, SoF = summary of findings.

The SoF Table (Fig. 7) presents the grade of the ultimate outcome under the intervention of D and F group with a result of no statistical significance and the “High” quality grade of this meta-analysis. According to the academic and clinical experiences, the grade of ultimate outcome and the overall grade quality of this meta-analysis, the grade strength of recommendation was “strong.”

## 4 Discussion

The debate on efficacy of decompression versus decompression plus fusion in lumbar spondylosis has never stopped and more intensified over several decades. Relevant publications insisted decompression alone to be significantly less invasive than that combined with fusion,^[26]^ Cassinelli et al^[27]^ demonstrated that posterior spinal fusion following decompression led to longer operative time, more blood loss, while instability of the spine is a potential consequence that needs to be considered,^[28,29]^ especially combined with DS. The recent publications included 3 RCTs^[14–16]^ focusing the issue, with more qualified and quantitative evidence, made it facilitate to perform a further study. Nine RCTs included in our meta-analysis showed there was no difference in the primary clinical outcomes as well as secondary ones of complications rate, reoperation rate ASD between D versus F while patients with fusion suffered more blood loss, prolonged operation time, and hospital stays. The study firstly based on all RCTs including newest publications, to perform a subgroup analysis and to show an evidence and recommendation grade.

Stability is an inevitable topic as a potential factors indicating the approach selection. Decompression alone was recommended for typical LSS with no lumber operation history, no spinal instability^[30]^ and decompression without fusion cannot guarantee consolidation as to the satisfactory outcomes.^[31]^ A survey^[5]^ reported that the presence of motion on dynamic radiographs and back pain might raise enough reason to choose fusion surgery. Herkowitz et al^[21]^ reported a difference on spondylolisthesis postoperatively between D and F group on flexion and extension position (5.8 mm vs 0.1 mm) and neutral position (7.9 mm vs 5.3 mm) (both *P* < .05 respectively) without significance preoperatively, while the DS progression postoperatively seemed not in line with the olishthesis degree in our analysis. Brown et al^[32]^ affirmed intraoperative spinal stiffness measurements did not predict clinical results after lumbar spine surgery. Försth et al^[14]^ also found no significant difference between the D and F groups in amelioration of back pain, regardless of DS, and previous studies have shown that spondylolisthesis was not associated with an increased level of back pain.^[33]^

In the last 3 years, more studies have approved that D alone was as effective as F for LS.^[34]^ In our meta-analysis, the primary outcomes deciding the majority efficacy such as the improment of VAS, ODI, and walking ability were of no difference, which was in array with some recent publications. Brodke et al^[35]^ reported fusion added to decompression had no superior survival curve, improved clinical outcomes over decompression alone. Although 3 different fusion with or without instruments as F group approaches included in our study, it concluded no significant differences were found in SF-36 and ODI score among 3 different fusion techniques for patients with DS and LSS.^[36]^ Therefore, an explanation for the result drawn by Ghogawala et al^[15]^ that F group was with slightly greater improvement in SF-36 than D alone statistically may be a factual clinical outcome but the overall main measures of no difference should be paid more attention. Spinal fusion surgery theoretically requires more intervention produces and often involves spinal implants or intervertebral cages,^[37]^ the secondary measures of operation duration, blood loss, and hospital stays were unquestionably less in D group though a various value in different studies, in agreement with most articles. As a consequence, we believed that D alone could achieve paralleled clinical efficacy compared with F approach.^[10]^ However, a reasonable selection should be required individually, when LS mixed with other degenerative changes, such as osteophyte or calcified ligaments, the more consolidation would make it possible to reduce a fusion. Matsudaira et al suggested a better clinical results outcome with DS by preserving the posterior elements.^[38]^ Similarly, Tuli et al thought the best alternative of adequate laminectomy with preservation of the posterior ligament complex integrity.^[39]^

The presence of DS has often been considered an instability, although there is no consensus on the definition^[14]^ and surgical strategies for DS was still a matter of debate. Six out of 9 RCTs referred to DS and there was a similar outcome as the whole meta-analysis when stratified for further subgroup analysis. McCullen et al proposed patients with DS may require changes in decompression without fusion modality to improve outcomes.^[40]^ Several studies suggested that decompression alone may exacerbate instability and increase the degree of DS.^[7,41,42]^ While Försth et al proved that F did not result in clinical outcomes that were superior to D with DS and our meta-analysis based on 2 studies about the number of postoperative DS progression showed no difference. Except the probability of major proportion the participants with DS took, the better explanation was there, factually, was of no significance between D and F group.

Long-term follow-up between the 2 approaches suggested no influence on clinical outcome.^[26]^ Follow-up time was always distinguished by 2 years and 5 years to conform short-, middle-, and long-term, while short term (<36 months) and middle-to-long term (>36 months) in this subgroup analysis performed as a reasonable combination of proportional distribution statistically and clinical sense. The change of ODI in middle-to-long term was of statistical difference but probably a bias as the little sample and significant heterogeneity (*I*^2^ > 50%), while that the reoperation rate in D group was higher (>36 months) may be make sense with the reason of ASD, which; however, was against the opinion of Inui et al^[20]^ that there was a significantly higher reoperation rate in fusion compared with decompression alone. In addition, Försth et al^[11]^ and Karlsson et al^[23]^ progressed the follow-up time of 5 years and refreshed information about some measures published in 2017, which, regretly, just contain a partial desirable results and eventually abandoned with an exclude study. It reported several paralleled measures of no difference between D and F groups: the VAS change on back pain (2.8 vs 3.2), the VAS change on leg pain (3.1 vs 3.2), the change of ODI (26 vs 29), the number of satisfaction (74 vs 64), and restenosis (7 vs 1). There was eventually no significant clinical outcomes yet between D and F group 3 years later.

The complications contained surgery associated events such as dural rupture and other adverse such as pulmonary embolism and cardiac infarction.^[14]^ It was regret that a further analysis should be progressed according to the types of complications but failed to obtain the desirable data. The overall incidence of complications were 14.71% in D group and 14.49% in F group (with a range of 0%–42%). Publications reported a higher grade of spondylolisthesis and older age were believed to be the risk factors of higher complication rate^[3,43]^ but we could not draw the same conclusion. In this meta-analysis, we found that the complication rate and reoperation rate did not differ significantly between D and F groups, which was different from most previous studies.^[3]^ ASD was an unavoidable complication and in theory the altered biomechanical function of the spine, was compensated for by increased motion at the unfused segments, which then accelerated adjacent lumbar level fusion problems and produced back pain and leg pain.^[44,45]^ While on the contrary, there was a higher ASD incidence in D group and the favor of F group indicated ASD may not be associated with fusion but a natural progression in LS, a consistent point drawn by Pesce et al^[46]^ that ASD is a part of the natural history of cervical spondylosis a complication based on a RCTs of 10 years follow-up. It seemed that surgeons might improperly attribute ASD as the common reason for poor outcomes after fusion surgery.^[14]^

Inui et al^[20]^ shown that there was a significantly higher reoperation rate in fusion compared with decompression alone. Dailey et al^[47]^ thought reoperation rate at the surgical level or adjacent levels was not associated with D or F and reported a 13% reoperation rate, a proximity of 10.90% in D group and 5.71% in F group in our study and of no difference between the 2 groups. There were publications reported the common causes of reoperations in the D group were the same segmental herniation and restenosis, while in the F group were caused by implant-related problems and ASD^[6,21]^ Brodke et al reported the common reason for reoperation was due to symptomatic adjacent segment pathology whatever the approaches (D or F),^[38]^ which was an approval by the same result in this analysis.

A cost-effective analysis was not included for the restriction of RCTs that just 1 out of 9 studies described, which, emphasized by Försth et al,^[14]^ showed the mean direct costs of each procedure (mainly hospital costs, including surgery) were $6800 higher in the F group than in the D group because of the additional operating time, extended hospitalization, and cost of the implant. Hallett et al revealed a cost difference of approximately USD $6290 per patient for an additional fusion implant.^[48]^ Given the higher cost of adding fusion, D alone was believed to be more cost-effective than instrumented fusion for selected patients.

There are several limitations restrict the overall efficacy: first of all, fusion surgery consists of various types while PLIF was different from PLF or F alone in terms of surgical procedure, which was a limitation of this study. Then, 9 RCTs included in this study with a less participants contrast with some relevant publications, confined by the number of RCTs although the supported a quality guarantee and evidence strength. In addition, a somewhat unsatisfied result of quality assessment with some high-risk factors probably downregulate the grade of recommendation, since 3 RCTs are still of moderate risk of bias and most of them could not exert inadequate blinding so as to produce 15% overestimation of treatment effect. Besides, there is insufficient data of primary outcomes in walking ability, SF-36 and further information on DS. Finally, the lack of results on radiographic findings may make an effect on an objective evaluation.

## 5 Conclusions

Decompression plus fusion has no better clinical results than decompression alone in short-level LSS, regardless of the combination with spondylolisthesis or the follow-up time. Decompression with fusion has a longer duration of operation, more hospital stays, and more blood loss. According to the GRADE, the grade of this meta-analysis is of “High” quality, the grade strength of recommendation was “strong.”

## Acknowledgment

The authors acknowledge Houshan Lv who contributed towards the study by making substantial contributions to the design and the acquisition of data.

## Author contributions

**Conceptualization:** Shuai Xu, Yan Liang, Kaifeng Wang, Yalong Qian, Haiying Liu.

**Data curation:** Shuai Xu, Yan Liang, Kaifeng Wang, Yalong Qian, Haiying Liu.

**Formal analysis:** Shuai Xu, Yan Liang, Yalong Qian, Haiying Liu.

**Funding acquisition:** Shuai Xu.

**Investigation:** Shuai Xu.

**Methodology:** Shuai Xu, Zhenqi Zhu, Haiying Liu.

**Project administration:** Jinyu Wang, Yan Liang, Zhenqi Zhu, Haiying Liu.

**Resources:** Jinyu Wang, Yan Liang, Zhenqi Zhu, Haiying Liu.

**Software:** Yan Liang, Zhenqi Zhu.

**Supervision:** Zhenqi Zhu, Kaifeng Wang.

**Validation:** Jinyu Wang, Yan Liang, Kaifeng Wang, Haiying Liu.

**Visualization:** Kaifeng Wang, Yalong Qian, Haiying Liu.

**Writing – original draft:** Shuai Xu, Jinyu Wang, Kaifeng Wang, Yalong Qian.

**Writing – review and editing:** Shuai Xu, Yan Liang, Haiying Liu.

## Supplementary Material

Supplemental Digital Content

## Supplementary Material

Supplemental Digital Content

## Supplementary Material

Supplemental Digital Content

## Supplementary Material

Supplemental Digital Content

## Supplementary Material

Supplemental Digital Content
