# Supplementary figures and images for: Decompression with fusion is not in superiority to decompression alone in lumbar stenosis based on randomized controlled trials: A PRISMA-compliant meta-analysis
Source: Medicine (Baltimore). 2019 Nov 15;98(46):e17849. doi: 10.1097/MD.0000000000017849 (PMC6867750; doi:10.1097/MD.0000000000017849)

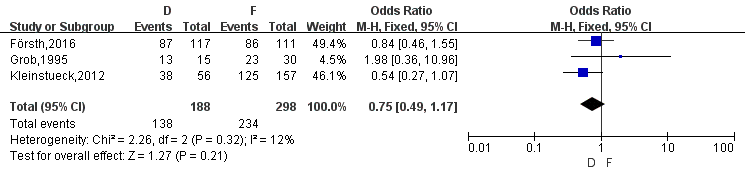


VAS decrease on back

Supplement: Supplemental Digital Content [file medi-98-e17849-s002.docx]

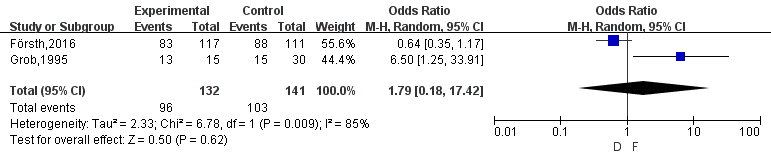


VAS decrease on leg

Supplement: Supplemental Digital Content [file medi-98-e17849-s003.docx]

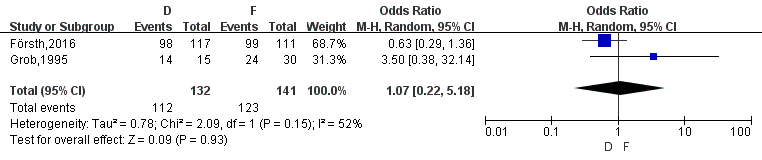


walking distance

Supplement: Supplemental Digital Content [file medi-98-e17849-s004.docx]

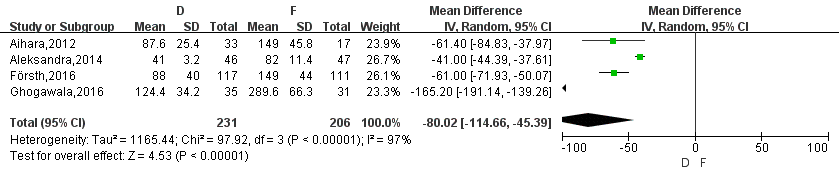


operation duration


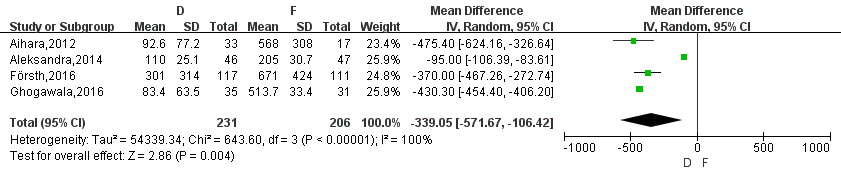


blood loss


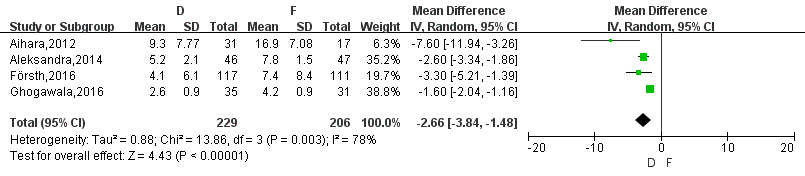


hospital stays

Supplement: Supplemental Digital Content [file medi-98-e17849-s005.docx]
